# Genetic analysis of porcine circovirus type 2 (PCV2) strains between 2002 and 2016 reveals PCV2 mutant predominating in porcine population in Guangxi, China

**DOI:** 10.1186/s12917-019-1859-z

**Published:** 2019-04-25

**Authors:** Jing Yao, Yanran Qin, Yue Zeng, Kang Ouyang, Ying Chen, Weijian Huang, Zuzhang Wei

**Affiliations:** Laboratory of Animal infectious Diseases and Molecular Immunology, College of Animal Science and Technology, Guangxi University, Nanning, 530005 People’s Republic of China

**Keywords:** PCV2, Genetic analysis, ORF2, Complete genome

## Abstract

**Background:**

Porcine circovirus 2-associated disease (PCVAD) is acknowledged as one of the most economically important diseases for the swine industry worldwide. The aim of this study was to characterize and determine the genetic diversity of PCV2 in the porcine population of Guangxi, China.

**Methods:**

The full length genome and open reading frame 2 (ORF2) of 95 PCV2 strains collected from the tissues and sera of pigs that had either died as a result of PCVAD or did not exhibit disease symptoms were analyzed.

**Results:**

The results of multiple sequence alignments showed that there is considerable diversity among the PCV2 ORF2 sequences. Phylogenetic analyses based on the complete genome showed that current PCV2 strains in this study could be divided into PCV2a (1/95), PCV2b (39/95), PCV2d (43/95), PCV2e (10/95) and PCV2h (2/95). Among the 5 sub-genotypes, PCV2b was dominant in the porcine population from 2002 to 2008. The newly identified sub-genotype, PCV2d, was seen from 2003 and has increased every year. PCV2b and PCV2d formed two predominant genetic groups circulating in southern China between 2009 and 2013 and the sub-genotype PCV2d has become the dominant virus in China since 2014.

**Conclusions:**

This study reveals the complex genetic diversity of PCV2 and improves our understanding regarding the epidemiological trends of PCV2 sub-genotypes in China.

**Electronic supplementary material:**

The online version of this article (10.1186/s12917-019-1859-z) contains supplementary material, which is available to authorized users.

## Background

Porcine circovirus 2 (PCV2) is the major etiological agent that causes PCV2-associated diseases (PCVAD) in growing pigs. This includes post-weaning multi-systemic wasting syndrome (PMWS), porcine dermatitis and nephropathy syndrome (PDNS), porcine respiratory disease complex (PRDC), congenital tremors type II (CT) and reproductive failure [1–4]. PCV2 is a small, single-stranded, non-enveloped, circular DNA virus containing a genome of 1766–1768 nt [5]. The PCV2 genome contains 11 open reading frames (ORFs) [5]. Five proteins, encoded by ORF1 to ORF5, are currently studied and recognized as the functional proteins of PCV2 [6–11]. Among these, the main ORF1 and ORF2 were identified as genes encoding viral replicase (Rep and Rep’) and capsid protein, respectively [6, 11]*.*

It has been shown that PCV2 is continuously evolving through point mutation and genome recombination, which can lead to some new antigenic variant strains and it is known that new PCV2 variant strains are emerging [12–14]. Phylogenetic analyses of the complete genome and ORF2 region of PCV2 isolates worldwide have shown that PCV2 could be divided into eight distinct genotypes. These have been named PCV2a, PCV2b, PCV2c, PCV2d, PCV2e, PCV2f, PCV2g and PCV2h according to a new genotyping methodology protocol [15]*.* PCV2a, PCV2b and PCV2d have been circulating worldwide and shown to have five (2A–E), three (1A–C) and two sub-genotypes (2d-1 and 2d-2) [13, 16], respectively, while the presence of PCV2c has only been reported in Denmark and Brazil [16–18]. PCV2e has been identified in pigs from China, Thailand, USA and Mexico [19–22]. Amongst all PCV2 genotypes, PCV2a was the predominant strain prior to 2000 and then there appeared to be a global genetic shift from PCV2a to PCV2b with the latter being the predominant genotype seen in the past ten years [18, 19, 23, 24]. Recently, there is a number of reports which suggest that there is an ongoing genotype shift occurring from PCV2b to PCV2d [13, 25]. In 2010, a variant PCV2 mutant strain designated mPCV2b, now grouped in PCV2d, with an elongation of its ORF2 by one amino acid, lysine (K), was identified in several PCVAD cases in China and other countries and recent studies showed that the prevalence rate of mPCV2b appears to have increased in China and a similar trend is evident in the U.S.A [13, 23, 26, 27].

Although there is increasing use of killed or subunit vaccines against PCV2 in pigs, the prevalence of PCV2 in China is still on the rise. Guangxi Province is one of the biggest pig breeding regions in China. The aim of this study was to investigate the prevalence and genetic variation of PCV2 in China using strains observed in the pig population from 2002 to 2016. Our findings revealed that PCV2d has becoming the predominating virus since 2014. Overall, this study helps to elucidate important aspects of the molecular genetic evolution of PCV2 and this is a prerequisite for the future development of effective disease control and prevention strategies for the spread of this virus.

## Results

### Prevalence of PRRSV in Guangxi Province, China from 2002 to 2016

Of the 371 filed samples collected from the clinical diseased and health pigs between 2002 and 2016 in the Guangxi Province of China, 181 samples (48.8%) were positive for PCV2, as determined by specific PCR. These results indicate that PCV2 is distributed widely among swine populations in the Guangxi Province.

### Sequence and phylogenetic analyses of the ORF2 gene of PCV2

To explore the genetic relationship and evolution of PCV2 from 2002 to 2016, 95 of 181 PCV2 positive samples were used for genome amplification and sequencing and phylogenetic analysis was carried out based on the sequences of the ORF2 gene of 95 PCV2 isolates with reference sequences. The results showed that the complete genomes of all 95 strains were 1767 or 1768 bp in length as shown in Table 1. Forty of the 95 ORF2 nucleotide sequences were 702 bp in length, encoding a Cap protein of 233 amino acid residues. Forty five of the 95 ORF2 nucleotide sequences were 705 bp, encoding a Cap protein of 234 amino acid residues. These strains are also known as mutant PCV2 (mPCV2), which has a codon shift from TTA to CTT in ORF2, resulting in a mutation of the stop codon (from UAA to AAG) in the ORF2, leading to an extended lysine (K) residue encoded by AAG or AAA.

**Table 1:** The designations, clinical signs, genotypes, GenBank accession numbers and other characteristics of the PCV2 genomes sequenced in this study

	Designation	Geographic origin	Clinical history	Tissue	Year of the collection	Genotype	GenBank No	Genome size (nt)	ORF2
1	GXNN0201	Nanning	PMWS	Inguinal lymph node	2002	PCV2b-1B	MH465415	1767	702
2	GXGG0201	Guigang	PMWS	Inguinal lymph node	2002	PCV2e	MH465483	1768	702
3	GXNN0202	Nanning	PMWS	Inguinal lymph node	2002	PCV2b-1A	MH465416	1767	702
4	GXNN0203	Nanning	PMWS	Inguinal lymph node	2002	PCV2b-1B	MH465417	1767	702
5	GXBH0301	Beihai	PMWS	Inguinal lymph node	2003	PCV2b-1A	MH481748	1767	702
6	GXNN0301	Nanning	PMWS	Inguinal lymph node	2003	PCV2d	MH465457	1767	705
7	GXYL0601	Yulin	PMWS	Inguinal lymph node	2006	PCV2b-1B	MH465433	1767	702
8	GXWZ0602	Wuzhou	PMWS	Inguinal lymph node	2006	PCV2b-1A	MH465432	1767	702
9	GXNN0603	Nanning	PMWS	Inguinal lymph node	2006	PCV2b-1B	MH465418	1767	702
10	GXHZ0708	Hezhou	PMWS	Inguinal lymph node	2007	PCV2b-1B	MH465407	1767	702
11	GXHZ0709	Hezhou	PMWS	Inguinal lymph node	2007	PCV2b-1B	MH465408	1767	702
12	GXHZ0710	Hezhou	PMWS	Inguinal lymph node	2007	PCV2b-1A	MH465409	1767	702
13	GXBH0801	Beihai	PMWS	Inguinal lymph node	2008	PCV2b-1B	MH465398	1767	702
14	GXLB0802	Wuxuan	PMWS	Inguinal lymph node	2008	PCV2d	MH465449	1767	705
15	GXGG0802	Guigang	PMWS	Inguinal lymph node	2008	PCV2b-1B	MH465404	1767	702
16	GXNN0803	Nanning	PMWS	Inguinal lymph node	2008	PCV2b-1A	MH465419	1767	702
17	GXNN0804	Nanning	PMWS	Inguinal lymph node	2008	PCV2d	MH465458	1767	705
18	GXCZ0805	Chongzuo	PMWS	Inguinal lymph node	2008	PCV2b-1B	MH465402	1767	702
19	GXGG0805	Guigang	PMWS	Inguinal lymph node	2008	PCV2d	MH465444	1767	705
20	GXNN0806	Nanning	PMWS	Inguinal lymph node	2008	PCV2b-1B	MH465420	1767	702
21	GXNN0901a	Nanning	No signs	Inguinal lymph node	2009	PCV2b-1B	MH465421	1767	702
22	GXNN0901b	Nanning	No signs	Inguinal lymph node	2009	PCV2d	MH465459	1767	705
23	GXNN0902	Nanning	No signs	Inguinal lymph node	2009	PCV2b-1B	MH465422	1767	702
24	GXNN0904	Nanning	Abortion	Aborted fetus	2009	PCV2d	MH465460	1767	705
25	GXBH1008	Beihai	PMWS	Inguinal lymph node	2010	PCV2b-1B	MH465399	1767	702
26	GXLZ1103a	Liuzhou	No signs	Inguinal lymph node	2011	PCV2b-1B	MH465414	1767	702
27	GXLZ1103b	Liuzhou	No signs	Inguinal lymph node	2011	PCV2d	MH465452	1767	705
28	GXLZ1208a	Liuzhou	No signs	Inguinal lymph node	2012	PCV2h	MH465453	1767	705
29	GXYL1208	Yulin	No signs	Inguinal lymph node	2012	PCV2h	MH465473	1767	705
30	GXLB1212a	Laibin	No signs	Inguinal lymph node	2012	PCV2e	MH465485	1768	702
31	GXLB1212b	Laibin	No signs	Inguinal lymph node	2012	PCV2d	MH465450	1767	705
32	GXLB1212c	Laibin	No signs	Inguinal lymph node	2012	PCV2e	MH465486	1768	702
33	GXGG1212	Guigang	No signs	Inguinal lymph node	2012	PCV2d	MH465405	1768	702
34	GXLZ1208b	Liuzhou	No signs	Inguinal lymph node	2012	PCV2d	MH465454	1767	705
35	GXLZ1208c	Liuzhou	No signs	Inguinal lymph node	2012	PCV2d	MH465455	1767	705
36	GXGG1208	Guigang	PMWS	Inguinal lymph node	2012	PCV2e	MH465484	1768	702
37	GXNN1209a	Nanning	PMWS	Inguinal lymph node	2012	PCV2b-1B	MH465423	1767	702
38	GXNN1209b	Nanning	PMWS	Inguinal lymph node	2012	PCV2b-1B	MH465424	1767	702
39	GXYL1304	Yulin	PMWS	Inguinal lymph node	2013	PCV2b-1B	MH465434	1767	702
40	GXNN1304a	Nanning	PMWS	Inguinal lymph node	2013	PCV2d	MH465461	1767	705
41	GXNN1304b	Nanning	PMWS	Inguinal lymph node	2013	PCV2b-1B	MH465425	1767	702
42	GXGG1305	Guigang	PMWS	Inguinal lymph node	2013	PCV2b-1B	MH465406	1767	702
43	GXYL1305	Yulin	PMWS	Inguinal lymph node	2013	PCV2b-1B	MH465435	1767	702
44	GXGG1306	Guigang	PMWS	Inguinal lymph node	2013	PCV2d	MH465445	1767	705
45	GXYL1307a	Yulin	PMWS	Inguinal lymph node	2013	PCV2d	MH465474	1767	705
46	GXYL1307b	Yulin	PMWS	Inguinal lymph node	2013	PCV2e	MH465490	1768	702
47	GXYL1307c	Yulin	PMWS	Inguinal lymph node	2013	PCV2d	MH465475	1767	705
48	GXYL1307d	Yulin	PMWS	Inguinal lymph node	2013	PCV2b-1B	MH465436	1767	702
49	GXYL1310	Yulin	PMWS	Inguinal lymph node	2013	PCV2b-1B	MH465437	1767	702
50	GXNN1312	Nanning	PMWS	Inguinal lymph node	2013	PCV2b-1B	MH465426	1767	702
51	GXGG1312a	Guigang	PMWS	Inguinal lymph node	2013	PCV2d	MH465446	1767	705
52	GXGG1312b	Guigang	PMWS	Inguinal lymph node	2013	PCV2d	MH465447	1767	705
53	GXBS1401	Baise	PMWS	Inguinal lymph node	2014	PCV2d	MH465439	1767	705
54	GXYL1401	Yulin	PMWS	Inguinal lymph node	2014	PCV2d	MH465476	1767	705
55	GXYL1403a	Yulin	PMWS	Inguinal lymph node	2014	PCV2d	MH465477	1767	705
56	GXYL1403b	Yulin	PMWS	Inguinal lymph node	2014	PCV2d	MH465478	1767	705
57	GXYL1405	Yulin	PMWS	Inguinal lymph node	2014	PCV2d	MH465479	1767	705
58	GXLB1405	Laibin	PMWS	Inguinal lymph node	2014	PCV2b-1B	MH465410	1767	702
59	GXLZ1406	Liuzhou	PMWS	Inguinal lymph node	2014	PCV2d	MH465456	1767	705
60	GXNN1406	Nanning	PMWS	Inguinal lymph node	2014	PCV2b-1B	MH465427	1767	702
61	GXYL1408	Yulin	PMWS	Inguinal lymph node	2014	PCV2e	MH465491	1767	705
62	GXNN1409a	Nanning	PMWS	Inguinal lymph node	2014	PCV2d	MH465462	1767	705
63	GXNN1409b	Nanning	PMWS	Inguinal lymph node	2014	PCV2e	MH465487	1768	702
64	GXYL1409	Yulin	PMWS	Inguinal lymph node	2014	PCV2b-1B	MH465438	1767	702
65	GXYL1410	Yulin	PMWS	Inguinal lymph node	2014	PCV2d	MH465480	1767	705
66	GXNN1410a	Nanning	PMWS	Inguinal lymph node	2014	PCV2d	MH465463	1767	705
67	GXCZ1410	Chongzuo	PMWS	Inguinal lymph node	2014	PCV2b-1B	MH465403	1767	702
68	GXNN1410b	Nanning	PMWS	Inguinal lymph node	2014	PCV2e	MH465488	1768	702
69	GXNN1410c	Nanning	PMWS	Inguinal lymph node	2014	PCV2d	MH465464	1767	705
70	GXBS1410	Baise	PMWS	Inguinal lymph node	2014	PCV2d	MH465440	1767	705
71	GXFC1501	Fangchenggang	PMWS	lymph node	2015	PCV2d	MH465443	1767	705
72	GXNN1501	Nanning	No signs	Lung, spleen, lymph node	2015	PCV2d	MH465465	1767	705
73	GXNN1503	Nanning	No signs	Lung, spleen, lymph node	2015	PCV2d	MH465466	1767	705
74	GXNN1504	Nanning	No signs	Lung, spleen, lymph node	2015	PCV2d	MH465467	1767	705
75	GXCZ1510a	Chongzuo	PMWS	lymph node	2015	PCV2d	MH465441	1767	705
76	GXCZ1510b	Chongzuo	PMWS	lymph node	2015	PCV2d	MH465442	1767	705
77	GXHC1511	Hechi	No signs	Lung, spleen, lymph node	2015	PCV2d	MH465448	1767	705
78	GXNN1511	Nanning	No signs	Lung, spleen, lymph node	2015	PCV2b-1B	MH465428	1767	702
79	GXLB1511a	Laibin	No signs	Lung, spleen, lymph node	2015	PCV2b-1B	MH465411	1767	702
80	GXLB1511b	Laibin	No signs	Lung, spleen, lymph node	2015	PCV2b-1B	MH465412	1767	702
81	GXLB1511c	Laibin	PMWS	Lung, spleen, lymph node	2015	PCV2b-1B	MH465413	1767	702
82	GXYL1512	Laibin	PMWS	Lung, spleen, lymph node	2015	PCV2d	MH465481	1767	705
83	GXQZ1601	Qinzhou	PMWS	lymph node	2016	PCV2d	MH465472	1767	705
84	GXNN1602	Nanning	PMWS	Lung, lymph node	2016	PCV2d	MH465468	1767	705
85	GXNN1603a	Nanning	PMWS	lymph node	2016	PCV2d	MH465469	1767	705
86	GXNN1603b	Nanning	PMWS	Lung	2016	PCV2b-1B	MH465429	1767	702
87	GXNN1604a	Nanning	No signs	Lung, spleen, lymph node	2016	PCV2a	MH465489	1768	702
88	GXNN1604b	Nanning	No signs	Lung, spleen, lymph node	2016	PCV2b-1B	MH465430	1767	702
89	GXLB1606	Laibin	PMWS	Lung, spleen, lymph node	2016	PCV2d	MH465451	1767	705
90	GXBS1607a	Baise	PMWS	Lung, spleen, lymph node	2016	PCV2e	MH465400	1767	702
91	GXBS1607b	Baise	PMWS	Lung, spleen, lymph node	2016	PCV2e	MH465401	1767	702
92	GXYL1607	Yulin	PMWS	Lung, spleen, lymph node	2016	PCV2d	MH465482	1767	705
93	GXNN1612a	Nanning	PMWS	spleen	2016	PCV2d	MH465470	1767	705
94	GXNN1612b	Nanning	No signs	lymph node	2016	PCV2d	MH465471	1767	705
95	GXNN1612c	Nanning	No signs	Lung, spleen, lymph node	2016	PCV2b-1B	MH465431	1767	702

**Fig. 1:**
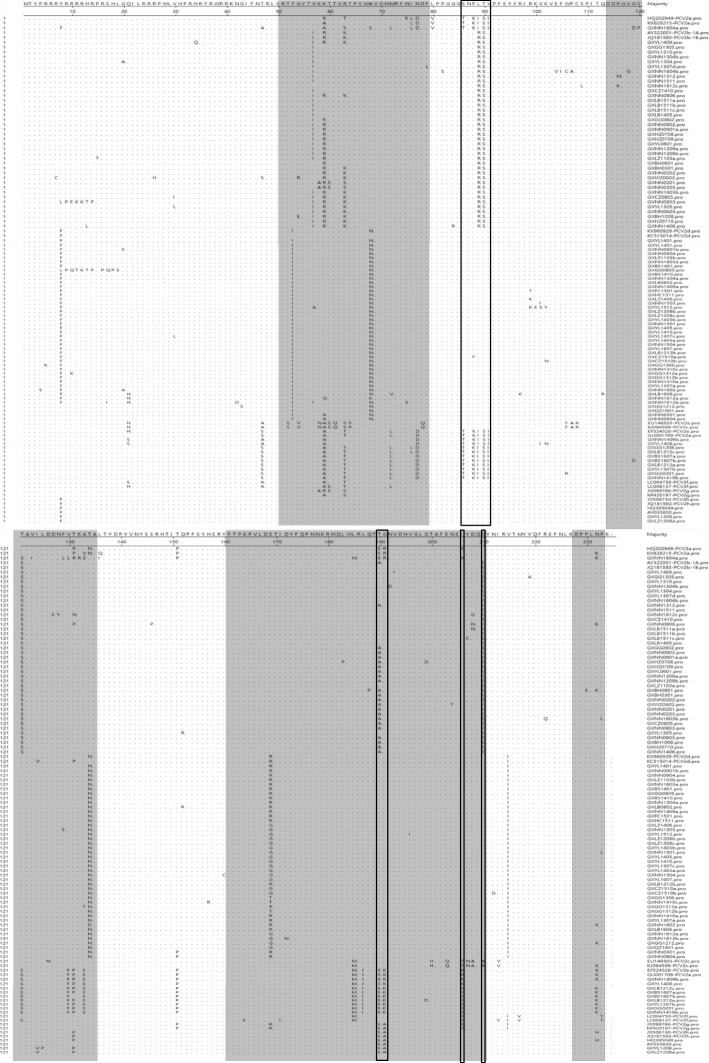
Phylogenetic tree based on a comparison of 129 complete PCV2 genomic sequences, including the 95 sequences from this study and 34 PCV2 sequences originating from China and other countries. The tree was constructed using the Maximum Likelihood algorithm. The 34 reference strains which are representatives of all PCV2 genotypes are marked with a black circle

Comparisons of the complete genomic sequence revealed 96.6% identity between PCV2a strains and the reference PCV2a strains (Table 2). The nucleotide sequence identity between PCV2b strains and reference PCV2b strains was 97.0–99.4%. The nucleotide sequence identity between PCV2d strains and the reference PCV2d strains was 97.1–99.9% and the nucleotide sequence identity between PCV2e strains and the reference PCV2e strains was 97.6~99.4% (Table 2). To investigate variations in the deduced amino acid sequences of ORF2 gene products, the amino acid sequences of 95 PCV2 strains including some representative strains were aligned. The results showed that there are five major regions of variation among the PCV2 strains. These include residues 57–91, 121–151, 181–191, 206–215 and 230–233 (Fig.1). One of the 96 strains has a typical TNKISI motif present in PCV2a. 39 of the 96 strains have typical S/PNPRSV and A/TGIE motifs present in PCV2b and 43/95 strains have SNPLTV and TGID motifs present in most of the PCV2d. PCV2e strains have a typical TNKISI motif which are also present in the PCV2a strains. Compared with PCV2a, PCV2e have specific substitutions at positions 47 (T to S), 72 (R to L), 131 (P to F), 187 (L to I) and 191(R to K). PCV2h strains have a typical SNPLTV motif which is present in most of the PCV2d strains. But the motif, TGID, was changed to SAID. Specific aa changes in the reported antibody epitope regions and immune-dominant decoy epitope regions (57–91, 181–191 and 230–233) of the Cap protein were found in some strains. Moreover, specific aa changes at positions 133–135 were also identified in some strains. As a result of a mutation at the stop codon, 45 of the 96 strains had an extended lysine (K) residue encoded by AAG or AAA.

**Table 2:** Comparison of the complete genomic sequences of the different PCV2 strains examined in this study

	KX828215 (PCV2a)	New strain (PCV2a)	AY916791 (PCV2b)	New strains (PCV2b)	KJ187306 (PCV2d)	New strains (PCV2d)	EF524526 (PCV2e)	New strains (PCV2e)	JX506730 (PCV2h)	New strains (PCV2h)	EU148503 (PCV2c)	LC004750 (PCV2f)	JX099786 (PCV2g)
KX828215 (PCV2a)	100.0	96.6	95.2	95.1~95.5	94.9	94.5~95.4	96.4	95.3~96.7	96.0	95.9~96.0	94.1	95.1	95.3
New strain (PCV2a)		100.0	94.6	94.4~95.2	94.2	93.7~94.5	96.6	95.2~96.9	95.4	95.2	93.2	95.1	94.1
AY916791 (PCV2b)			100.0	97.0~99.4	95.9	95.4~96.3	95.5	93.9~95.7	96.4	96.1~96.2	94.8	95.6	95.5
New strains (PCV2b)				97.1~100.0	95.9~97.2	95.1~97.3	95.2~95.9	93.9~97.2	96.0~96.7	96.0~97.2	94.5~95.3	95.4~96.0	95.5~97.2
KJ187306 (PCV2d)					100.0	97.9~99.7	95.0	94.2~95.3	97.6	96.6~96.7	94.5	95.5	96.6
New strains (PCV2d)						97.1~99.8	94.3~95.4	93.2~95.7	96.0~96.9	96.0~97.1	93.9~94.9	95.0~95.9	96.2~97.0
EF524526 (PCV2e)							100.0	97.6~99.4	95.9	95.5~95.6	94.1	96.1	94.7
New strains (PCV2e)								96.8~99.7	94.5~96.3	94.9~96.0	92.8~94.2	94.9~97.4	93.8~95.0
JX506730 (PCV2h)									100.0	98.4	95.0	96.0	95.8
New strains (PCV2h)										99.7	94.8~94.9	96.1~96.2	96.8~96.9
EU148503 (PCV2c)											100.0	94.7	94.8
LC004750 (PCV2f)												100.0	95.5
JX099786 (PCV2g)													100.0

**Fig. 2:**
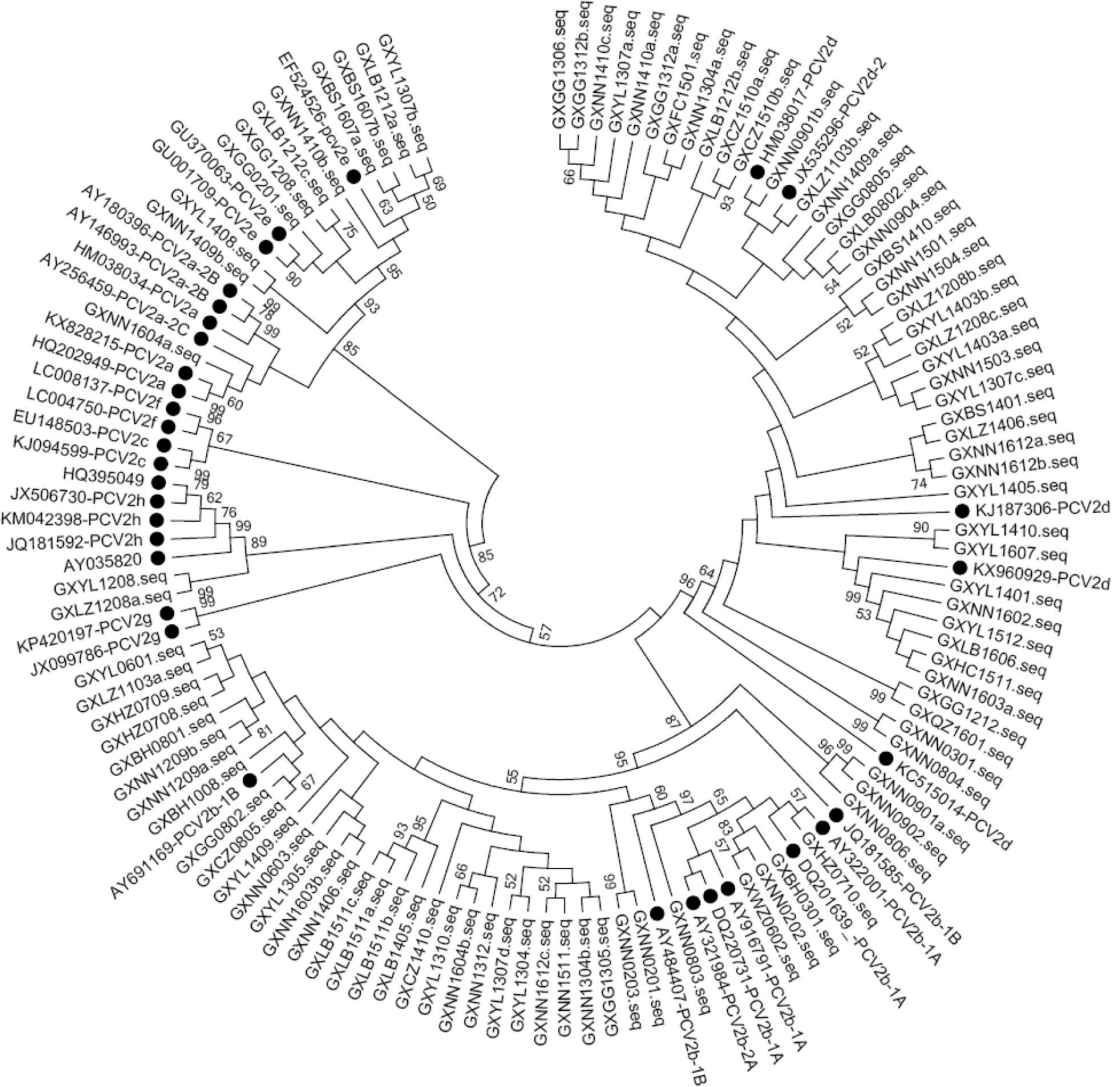
The alignment of Cap for PCV2. A multiple alignment of PCV2 Cap was performed by Clustal W. The grey areas show the antibody recognition domains and the immune-dominant decoy epitope described previously [30, 31]. The boxes show the motifs of PCV2a, PCV2b and PCV2d which have been previously described [13, 28, 29]

**Fig. 3:**
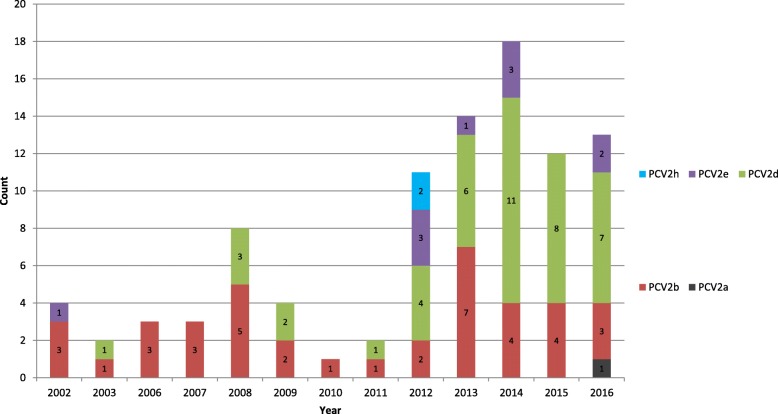
The time distribution and genotype of the 95 PCV2 strains in this study

Phylogenetic analysis of the complete genome showed that current PCV2 strains in this study could be divided into PCV2a (1/95), PCV2b (39/95), PCV2d (43/95), PCV2e (10/95) and PCV2h (2/95), as shown in Fig. 2. The genotype PCV2b was further divided into PCV2b-1A (5/95) and PCV2b-1B (34/95). All 95 strains with a complete genome phylogeny have the same classification with respect to the ORF2-based phylogeny, except for two strains (GXNN0301and GXNN0604) which were clustered to PCV2g (Additional file 1: Figure S1). Among the 5 sub-genotypes, PCV2b was dominant in the porcine population from 2002 to 2008. The newly identified sub-genotype, PCV2d, was found from 2003 and its presence has increased year by year. PCV2b and PCV2d are two predominant genetic groups which circulated in the Guangxi Province between 2009 and 2013 and PCV2d is the predominant genotype circulating in the swine population of this region since 2014 (Fig. 3).

## Discussion

In our previous study, 181 of the 371 (48.8%) samples collected were positive for PCV2, indicating that PCV2 is widely distributed among swine populations in Guangxi, China. There is extensive genetic variability in four major regions at amino acid positions 53–90, 121–136, 169–218 and 232–234. There are critical aa’s within some signature motifs which are reported to be important for differentiation of the PCV2 genotype [13, 28, 29] as well as regions such as antibody epitopes, immune-dominant decoy epitopes and key aa’s which determine virulence [30–34] which were found in the Cap protein domains of some strains. ORF2 is the major structural protein of PCV2 that is believed to be involved in diverse functions such as receptor binding, host immune response and viral replication [6, 32–34]. Therefore, a small number of mutations might result in antigenic variations or increased pathogenicity of the virus.

Full length genome and ORF2 based phylogenetic trees showed all these strains are present in 5 sub-genotypes (PCV2a, PCV2b, PCV2d, PCV2e and PCV2h). Global genetic analysis indicated that the PCV2 evolution trace was PCV2a to PCV2b to PCV2d [13]. Many previous studies showed that genotype shift from PCV2a to PCV2b occurred in 2002 in mainland China and PCV2b has been the predominant genotype since then [19, 23, 24]. A similar major shift from PCV2a to PCV2b has also occurred in many countries on a global scale prior to 2003 [35, 36]. Consistent with these previous studies, our study shows that only one strain (PCV2a) was found in 2016, suggesting a major shift from PCV2a to PCV2b had occurred in Guangxi Province in or prior to 2002. The PCV2b is the predominant genotype found between 2003 and 2011.

Many studies have indicated that the rapid genotype shift from PCV2a to PCV2b was related to the appearance of PMWS cases at the farmyard level together with an accompanying increase in clinical severity [37–39]. However, there are no significant difference in virulence between PCV2a and PCV2b-inoculated groups under experimental conditions [40, 41]. In this study, we also showed there was no significant relationship between the infection caused by PCV2b and PMWS cases (data not shown). Both PCV2a and PCV2b could be detected in the healthy pigs and in PWMS-affected pigs (Table 1). Our results showed that PCV2b and PCV2d were the two predominant genetic groups circulating in southern China between 2008 and 2013. PCV2d was the predominant genotype seen since 2014, indicating that the process leading to the genotype shift from PCV2b to PCV2d had already begun at the province-wide scale in these subsequent years.

In 2010, a variant PCV2 mutant strain designated as mPCV2b, and now classified as PCV2d, was identified and the prevalence rate of mPCV2b appears to have increased both in China and the USA. In this study, a dramatic increase in detection of the PCV2d variant has been seen since 2014. Whether the PCV2d is more pathogenic in pigs is controversial. One study conducted by Guo et al. showed that mPCV2, now classified as PCV2d, induced more severe clinical, pathological, and virological manifestations than the genotypes PCV2a and PCV2b in conventional pigs [23]. However, another study showed that there was no significant difference in pathogenicity between PCV2a/b and mPCV2 in caesarean-derived, colostrum-deprived pigs [42].

In this study, we showed there was no significant relationship between the predominance of PCV2d and PMWS cases. Therefore, the pathogenicity of mPCV2 in pigs and the association between increased mPCV2 prevalence and its clinical manifestation in the field needs to be further studied.

## Conclusions

This study reveals the complex genetic diversity of PCV2 and improves our understanding regarding the epidemiological trends of PCV2 sub-genotypes in China.

## Methods

### Sample collection, viral DNA extraction and PCV2 detection

Field samples (sera, lungs, lymph nodes and spleens) from commercial pig farms in different regions of Guangxi province between 2002 and 2016 were submitted to Laboratory of Animal infectious Diseases and Molecular Immunology, Guangxi University, Nanning for PCV2 testing. Total viral DNA was extracted directly from sera and tissue samples using Virus Genome Extract DNA kit according to the manufacturer’s instructions (TIANGEN, Inc., Beijing, China). Viral DNAs were eluted in 50 μL of ddH_2_O and were stored at − 30 °C until used. All the samples were screened for PCV2 by PCR using primers (5′-CCGCGGGCTGGCTGAACTT-3′) and (5′-ACCCCCGCCACCGCTACC -3′). Thermal cycling conditions were 94 °C for 3 min, followed by 35 cycles of 94 °C for 40 s, 60 °C for 40s, 72 °C for 50 s, and a final elongation step at 72 °C for 10 min. Finally, the PCR products were analyzed using 1.0% agarose gel electrophoresis ultraviolet imaging. Positive samples were determined with 1154 bp amplified products. Positive amplicons were purified using E.Z.N.A.TM Gel Extraction Kit (OMEGA, USA) and were further cloned into pBST-IIvector (TIANGEN, Inc., Beijing, China) for nucleotide sequencing by using primer T_7_ or T_3_ (HuaDa Gene, Inc., China).

### PCV2 amplification and sequence determination

PCV2 positive samples were used for full-length genome amplification and sequencing. The forward primer (5′ GAACCGCGGGCTGGCTGAACTTTTGAAAGT 3′) and reverse primer (5′ GCACCGCGGAAATTTCTGACAAACGTTACA 3′) were used for amplification of the full genome. PCR reaction conditions were 94 °C for 5 min, followed by 30 cycles of 94 °C for 1 min, 55 °C for 1 min, 72 °C for 2 min, and a final elongation step at 72 °C for 10 min. The PCR products were purified with E.Z.N.A.TM Gel Extraction Kit (OMEGA, USA) and cloned into a *pBST-II*vector (TIANGEN, Inc., Beijing, China). Positive clones were sequenced in both directions using universal primers T7 and SP6 promoter-specific primers (HuaDa Gene, Inc., China).

### Phylogenetic tree analysis

Differences of the amino acid sequences derived from the ORF2 gene of the 95 strains and representative isolates from China and other countries were analyzed and aligned using DNAstar software (DNASTAR Inc., Madison, WI, USA). MEGA version 6.0 was used to evaluate phylogenetic relationships by the Maximum Likelihood method with 1000 bootstrap replicates. A Maximum Likelihood phylogenetic tree was constructed including the 95 different complete genomes or ORF2 genes from this study and the complete genome or ORF2 gene sequences of the 34 representative isolates from China and other countries representative of all PCV2 genotypes, containing the considered PCV2 genotypes a, b, c, d, e, f, g and h. The sequences obtained in this study were submitted to the GenBank database under the accession numbers (MH465398~MH465491 and MH481748).

### Statistical analysis

PASW Statistics 18 software (PASW, Inc., an IBM Company, Chicago, IL) was used to perform χ_2_ test to evaluate the association of PCV2b and PCV2d with PMWS cases. *P* values of < 0.05 were considered statistically significant.

## Additional file

Additional file 1: Phylogenetic tree based on PCV2 ORF2 sequences**.** Phylogenetic tree based on a comparison of 129 PCV2 ORF2 sequences, including the 95 sequences from this study and 34 PCV2 sequences originating from China and other countries. The tree was constructed using the Maximum Likelihood algorithm. The 34 reference strains which are representatives of all PCV2 genotypes are marked with a black circle. (PPTX 203 kb)

## Abbreviations

CT: Congenital tremors type II
ORFs: Open reading frames
PCV2: Porcine circovirus type 2
PCVAD: PCV2-associated diseases
PDNS: Porcine dermatitis and nephropathy syndrome
PMWS: Post-weaning multi-systemic wasting syndrome
PRDC: Porcine respiratory disease complex
