# Supplementary figures and images for: Genetic analysis of porcine circovirus type 2 (PCV2) strains between 2002 and 2016 reveals PCV2 mutant predominating in porcine population in Guangxi, China
Source: BMC Vet Res. 2019 Apr 25;15:118. doi: 10.1186/s12917-019-1859-z (PMC6482503; doi:10.1186/s12917-019-1859-z)

## Slide 1
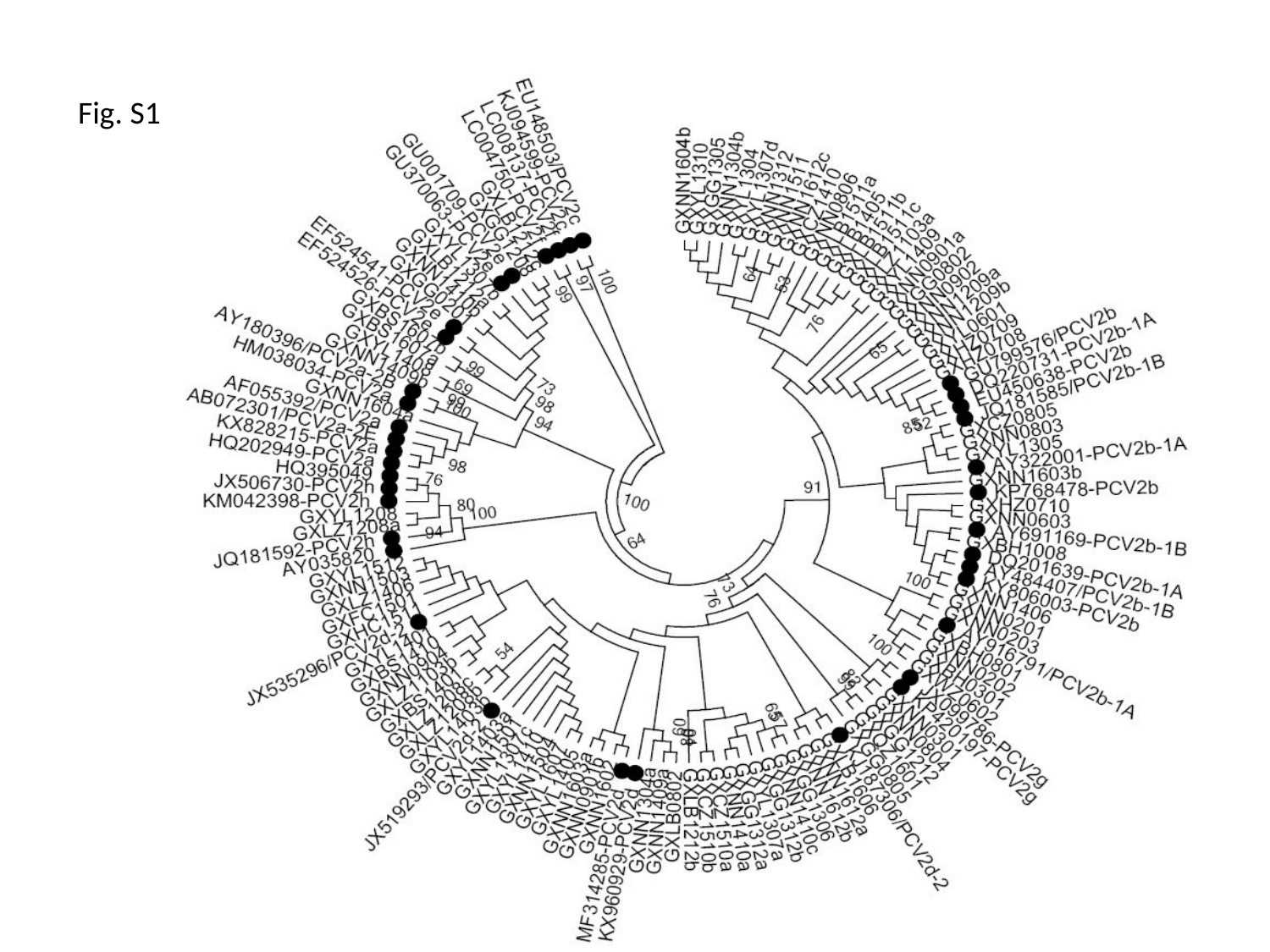

Fig. S1

Supplement: Supplementary file 1 — Phylogenetic tree based on PCV2 ORF2 sequences. Phylogenetic tree based on a comparison of 129 PCV2 ORF2 sequences, including the 95 sequences from this study and 34 PCV2 sequences originating from China and other countries. The tree was constructed using the Maximum Likelihood algorithm. The 34 reference strains which are representatives of all PCV2 genotypes are marked with a black circle. (PPTX 203 kb) [file 12917_2019_1859_MOESM1_ESM.pptx]
